# Thresholded Two-Phase Test Sample Representation for Outlier Rejection in Biological Recognition

**DOI:** 10.1155/2013/248380

**Published:** 2013-03-11

**Authors:** Xiang Wu, Ning Wu

**Affiliations:** ^1^Harbin Institute of Technology, 92 West Dazhi Street, Nan Gang District, Harbin 150001, China; ^2^Shenzhen Key Lab of Wind Power and Smart Grid, Harbin Institute of Technology Shenzhen Graduate School, Shenzhen 518055, China

## Abstract

The two-phase test sample representation (TPTSR) was proposed as a useful classifier for face recognition. However, the TPTSR method is not able to reject the impostor, so it should be modified for real-world applications. This paper introduces a thresholded TPTSR (T-TPTSR) method for complex object recognition with outliers, and two criteria for assessing the performance of outlier rejection and member classification are defined. The performance of the T-TPTSR method is compared with the modified global representation, PCA and LDA methods, respectively. The results show that the T-TPTSR method achieves the best performance among them according to the two criteria.

## 1. Introduction

Object recognition has become a hot topic in the field of computer vision and pattern recognition in recent years, and many approaches have been proposed for face image classification with a given database. One type of the methods is to reduce the dimensionality of sample by extracting the feature vector with linear transformation methods, such as the principal component analysis (PCA) [1–3] and the linear discriminant analysis (LDA) [4, 5]. In the PCA method, the training samples and the testing samples are transformed from the original sample space into a space with the maximum variance of all the samples, while the LDA method converts the samples to a feature space where the distances of the centers of different classes are maximized. In these two transformation methods, both the training samples and the testing samples have their corresponding representations in the new feature space, and the classification is carried out based on the distance between the representations related to the training set and the testing set.

 Another type of transformation-based method was proposed to focus on local information of the training samples. Instead of using the whole training set, this type of method only uses part of the samples, since the performance of the classifier is usually limited within some local areas. By concentrating on the local distribution of training data, the design and testing of the classifier can be much more efficient than the global methods [6]. Typical examples of local LDA methods include the method for multimodal data projection [7, 8] and the approach to use the local dependencies of samples for classification [9]. It is also found that the local PCA is more efficient than the global PCA in feature extraction [10] or sample clustering [11].

In recent years, the sparse representation theory has been applied to pattern recognition problems and has drawn a lot of attentions [12–21]. The sparse representation method also uses only part of the training data for classification by linearly representing a testing sample with the training set, and part of the linear combination coefficients is set to zero. The classification criterion of the sparse representation method is based on the biggest contribution from the sample classes during the linear representation.

In a recent study, a two-phase test sample representation (TPTSR) method was proposed for face recognition [22]. In this method, classification process is divided into two steps: the first step selects *M*-nearest neighbors of the testing sample from the training set by using linear representation method and the second step processes the selected *M* samples further by using them to linearly represent the testing sample. The classification result is based on the linear contribution of the classes among the *M*-nearest neighbors in the second phase of the TPTSR. By selecting *M*-closest neighbors from the training set for further processing, the TPTSR method identifies a local area that may contain the target class sample, reducing the risk of misclassification because of a similar nontarget sample.

Even the TPTSR method has been proven to be very useful in face classification; however, for face recognition applications with outliers the classification emphasis is different and the performance measurement criterion is also new. In face recognition problems with outliers, like security registration systems, only a small and particular group of members is required to be classified and compared with a large population of irrelevant people or intruders. In the application of identifying wanted criminals at airports, train station and other public places, the classifier is also required to identify a minor number of target members from a large number of irrelevant passengers. In previous studies, the approaches for pattern classification with outliers include two main methods, one is to train the classifier with only the member samples, and the other is to take into account a small number of outliers as a separate class in the training set [23]. However, neither of the methods can guarantee a low false alarm rate while maintaining a reasonable recognition rate for members.

In this paper, we further develop the TPTSR method by applying a threshold in the classification process for outlier rejection and member classification, and it is referred to as thresholded TPTSR (T-TPTSR) method. In the T-TPTSR, the distance between the testing sample and the weighted contribution of the target class in the second-phase linear representation is measured and compared with a threshold, by which an outlier will be identified. In this study, we also propose two different criteria for assessing the performance of classifier for outlier rejection as well as member classification, and, based on these criteria, we test the thresholded global representation (T-GR) method, thresholded PCA (T-PCA) method, and thresholded LDA (T-LDA) method, respectively. The test results show that the T-TPTSR achieves better performance in rejecting the outliers while maintaining outstanding classification rate for members.

In Sections 2 and 3 of this paper, we will introduce the theory of the T-TPTSR, T-GR, T-PCA, and T-LDA, respectively.  Section 4 presents our experimental results with different face image databases, and finally a conclusion will be drawn in Section 5.

## 2. Thresholded Two-Phase Test Sample Representation (T-TPTSR)

In this section, the TTPTSR method will be introduced with a threshold applied to the second-phase output in the classification process.

### 2.1. First Phase of the T-TPTSR with M-Nearest Neighbor Selection

 The first phase of the T-TPTSR is to select *M*-nearest neighbors from all the training samples for further processing in the second phase, narrowing the sample space down to a local area for the target class [22]. The *M*-nearest neighbors are selected by calculating the weighted distances of the testing sample from each of the training samples. Firstly, let us assume that there are *L* classes and *n* training images, *x*_1_, *x*_2_,…, *x*_*n*_, and if some of these images are from the *j*th class (*j* = 1, 2,…, *L*), then *j* is their class label. It is also assumed that a test image *y* can be written in the form of linear combination of all the training samples, such as

(1) $$\begin{array}{c} y=a_{1}x_{1}+a_{2}x_{2}+\cdots +a_{n}x_{n}, \end{array}$$

where *a*_*i*_ (*i* = 1, 2,…, *n*) is the coefficient for each training image *x*_*n*_. Equation (1) can also be written in the form of vector operation, such as

(2) $$\begin{array}{c} y=XA, \end{array}$$

where *A* = [*a*_1_⋯*a*_*n*_]^*T*^, *X* = [*x*_1_⋯*x*_*n*_]^*T*^.*x*_1_ ⋯ *x*_*n*_, and *y* are all column vectors. If *X* is a singular square matrix, (2) can be solved by using *A* = (*X* ^*T*^ *X*+*μI*)^−1^ *X* ^*T*^ *y*, or it can be solved by using *A* = *X* ^−1^ *y*, where *μ* is a small positive constant and *I* is the identity matrix. In our experiment with the T-TPTSR method, *μ* in the solution is set to be 0.01.

By solving (2), we can represent the testing image using the linear combination of the training set as shown in (1), which means that the testing image is essentially an approximation of the weighted summation of all the training images, and the weighted image *a*_*i*_ *x*_*i*_ is a part of the approximation. In order to measure the distance between the training image *x*_*i*_ and the testing image *y*, a distance metric is defined as followed:

(3) $$\begin{array}{c} e_{i}={||y-a_{i}x_{i}||}^{2}, \end{array}$$

where *e*_*i*_ is called the distance function, and it gives the difference between the testing sample *y* and the training sample *x*_*i*_. It is clear that a smaller value of *e*_*i*_ means that the *i*th training sample is closer to the testing sample, and it is more probable to be the member of the target class. These *M*-nearest neighbors are chosen to be processed further in the second phase of the T-TPTSR where the final decision will be made within a much smaller sample space. We assume that the *M*-nearest neighbors selected are denoted as *x*_1_ ⋯ *x*_*M*_, and the corresponding class labels are *C* = {*c*_1_ ⋯ *c*_*M*_}, where *c*_*i*_ ∈ {1, 2,…, *L*}. In the second phase of the T-TPTSR, if a sample *x*_*p*_'s class label does not belong to *C*, then this class will not be considered as a target class, and only a class from *C* will be regarded as a potential target class.

### 2.2. Second Phase of the T-TPTSR for Outlier Rejection

In the second phase of the T-TPTSR method, the *M*-nearest neighbors selected from the first phase are further calculated to obtain a final decision for the recognition task. We represent the testing sample with the linear combination of the training samples again, but only with the *M*-nearest neighbors selected from the first phase. If the *M*-nearest neighbors selected are denoted as *x*_1_ ⋯ *x*_*M*_, and their linear combination for the approximation of the testing image *y* is assumed to be satisfied, such as

(4) $$\begin{array}{c} y=b_{1}x_{1}+\cdots +b_{M}x_{M}, \end{array}$$

where *b*_*i*_ (*i* = 1, 2,…, *M*) are the coefficients. In vector operation form, (4) can be written as

(5) $$\begin{array}{c} y=\tilde{X}B, \end{array}$$

where *B* = [*b*_1_⋯*b*_*M*_]^*T*^, and $\tilde{X}=[x_{1}\cdots x_{M}]$. In the same philosophy as above, if $\tilde{X}$ is a nonsingular square matrix, (5) can be solved by

(6) $$\begin{array}{c} B={\left(\tilde{X}\right)}^{-1}y, \end{array}$$

or, otherwise, *B* can be solved by

(7) $$\begin{array}{c} B={\left({\tilde{X}}^{T}\tilde{X}+\gamma I\right)}^{-1}{\tilde{X}}^{T}y, \end{array}$$

where *γ* is a positive small value constant, and it is usually set to 0.01, and *I* is the identity matrix. 

When we obtain the coefficients *b*_*i*_ for each of the nearest neighbors, the contribution of each of the classes to the testing image will be measured, and the classification output will be based on the distance between the contribution and the testing image. If the nearest neighbors *x*_*s*_ ⋯ *x*_*t*_ are from the *r*th class (*r* ∈ *C*), and the linear contribution to approximate the testing sample by this class is defined as

(8) $$\begin{array}{c} g_{r}=b_{s}x_{s}+\cdots +b_{t}x_{t}. \end{array}$$

The measurement of the distance between the testing sample and the *r*th class samples in the *M*-nearest neighbors is calculated by the deviation of *g*_*r*_ from *y*, such as

(9) $$\begin{array}{c} D_{r}={||y-g_{r}||}^{2},\;r\in C. \end{array}$$

It is clear that a smaller value of *D*_*r*_ means a better approximation of the training samples from the *r*th class for the testing sample, and thus the *r*th class will have a higher possibility over other classes to be the target class. However, if outliers are considered, a threshold must be applied to the classification output to differentiate the members of class from outliers, such as

(10) $$\begin{array}{c} D_{k}=\min D_{r}<T\;\left(k,r\in C;T\in \left[0,+\infty \right)\right), \end{array}$$

where *T* is the threshold. If *D*_*k*_ ≥ *T*, the testing sample will be regarded as an outlier and therefore will be rejected. Only when *D*_*k*_ < *T*, the testing sample *y* can be classified to the *k*th class with the smallest deviation from *y*.

In the second phase of the T-TPTSR, the solution in (6) or (7) finds the coefficients for the linear combination of the *M*-nearest neighbors to approximate the testing sample, and the training class with the minimum deviation of the approximation will be considered as the target class for the testing sample. However, the value of the minimum deviation must be less than the threshold *T*. If the minimum distance between the testing sample and the member class's approximations is greater than the threshold *T*, the testing sample will be classified as an outlier and thus rejected. However, if the value of the minimum deviation of the linear combinations to an outlier is less than the threshold *T*, this outlier will be classified into the member class with the minimum deviation, and a misclassification will occur. Likewise, if a testing image belongs to a member class, but the minimum deviation from the linear combinations of each of the classes is greater than the threshold *T*, this testing image will be classified as an outlier, and a false alarm is resulted. Since the samples used in the T-TPTSR method are all normalized in advanced, the value of *D*_*r*_ in (9) will be within a certain range, such that 0 ≤ *D*_*r*_ ≤ *s*, where *s* ≈ 1, and therefore it is practical to determine a suitable threshold for the identification task before the testing.

## 3. The T-GR, T-PCA, and T-LDA Methods for Outlier Rejection

As a performance comparison with the T-TPTSR method, in the following section, we also introduce the modified versions of the GR, PCA, and LDA methods, respectively, for outlier rejection and member classification in face recognition.

### 3.1. The T-GR Method

The thresholded global representation (T-GR) method is essentially the T-TPTSR method with all the training samples that are selected as the *M*-nearest neighbors (*M* is selected as the number of all the training samples), and it also finds the target class directly by calculating the best representing sample class for the testing image. 

In the T-GR method, the testing sample is represented by the linear combination of all the training samples, and the classification is not just based on the minimum deviation of the linear contribution from each of the classes to the testing sample, but also based on the value of the minimum deviation. If the minimum deviation is greater than the threshold applied, the testing sample will be identified as an outlier.

### 3.2. The T-PCA Method

The PCA method is based on linearly projecting the image space onto a lower-dimensional feature space, and the projection directions are obtained by maximizing the total scatter across all the training classes [24, 25]. Again, we assume that there are *L* classes and *n* training images, *x*_1_, *x*_2_,…, *x*_*n*_, each of which is *m*-dimensional, where *n* < *m*. If a linear transformation is introduced to map the original *m*-dimensional image space into an *l*-dimensional feature space, where *l* < *m*, the new feature vector *u*_*i*_ ∈ *R* ^*l*^ can be written in the form of

(11) $$\begin{array}{c} u_{i}=W^{T}x_{i}\;\left(i=1,2,\ldots ,n\right), \end{array}$$

where *W* ^*T*^ ∈ *R* ^*m*×*l*^ is a matrix with orthonormal columns. If the total scatter matrix *S* ^*T*^ is defined as

(12) $$\begin{array}{c} S^{T}=\sum_{i=1}^{n}\left(x_{i}-\mu \right){\left(x_{i}-\mu \right)}^{T}, \end{array}$$

where *μ* ∈ *R* ^*m*^ is the mean of all the training samples, we can see that, after applying the linear transformation *W* ^*T*^, the scatter of all the transformed feature vectors *u*_1_, *u*_2_,…, *u*_*n*_ is *W* ^*T*^ *S* ^*T*^ *W*, which can be maximized by finding a projection direction *W*_*m*_, such as

(13) Wm=arg max⁡W WTSTW=[w1,w2⋯wl],

where *w*_*i*_ (*i* = 1,…, *l*) is the set of *m*-dimensional eigenvectors of *S* ^*T*^ corresponding to the *l* biggest eigenvalues. During the recognition process, both the testing sample *y* and all the training samples are projected into the new feature space via *W*_*m*_ before the distance between them is calculated, such as

(14) Di=||WmTy−WmTxi||2=||WmT(y−xi)||2 (i=1,2,…,n).

In the thresholded PCA method, the testing sample *y* will be classified to the class whose member has the minimum distance *D*_*i*_, but this distance must be less than the threshold *T*, such that

(15) $$\begin{array}{c} D_{k}=\min D_{i}<T\;\left(k,i=1,2,\ldots ,n;\;T\in \left[0,+\infty \right)\right). \end{array}$$

The testing sample *y* whose corresponding minimum distance *D*_*k*_ is less than the threshold *T* will be classified as an outlier and therefore rejected; otherwise *y* will be classified into the class with *x*_*k*_.

### 3.3. The T-LDA Method

The LDA is a class-specific linear method for dimensionality reduction and simple classifiers in a reduced feature space [26–29]. The LDA method also finds a direction to project the training images and testing images into a lower dimension space, on the condition that the ratio of the between-class scatter and the within-class scatter is maximized. 

Likewise, if there are *L* classes and *n* training images, *x*_1_, *x*_2_,…, *x*_*n*_, each of which is *m*-dimensional, where *n* < *m*, and in the *i*th class there are *N*_*i*_ samples (*i* = 1,2,…, *L*), the between-class scatter matrix can be written as

(16) $$\begin{array}{c} S_{b}=\sum_{i=1}^{L}N_{i}\left(\mu_{i}-\mu \right){\left(\mu_{i}-\mu \right)}^{T}, \end{array}$$

and the within-class scatter matrix can be defined as

(17) $$\begin{array}{c} S_{w}=\sum_{i=1}^{L}\sum_{j=1}^{N_{i}}\left(x_{j}-\mu_{i}\right){\left(x_{j}-\mu_{i}\right)}^{T}, \end{array}$$

where *μ*_*i*_ is the mean image of the *i*th class, and *μ* is the mean of all the samples. It is noted that *S*_*w*_ must be nonsingular in order to obtain an optimal projection matrix *W*_*m*_ with the orthonormal columns to maximize the ratio of the determinant of the projected *S*_*b*_ and projected *S*_*w*_, such that

(18) Wm=argmax⁡W|WTSbW|WTSwW=[w1w2⋯wl],

where *w*_*i*_ (*i* = 1,…, *l*) is the set of *m*-dimensional generalized eigenvectors of *S*_*b*_ and *S*_*w*_ corresponding to the *l* biggest eigenvalues, such as

(19) $$\begin{array}{c} S_{b}w_{i}=\lambda_{i}S_{w}w_{i},\;\left(i=1,2,\ldots ,l\right), \end{array}$$

where *λ*_*i*_ (*i* = 1,…, *l*) is the *l* generalized eigenvalues. Since there are the maximum number of *L* − 1 nonzero generalized eigenvalues available, the maximum *l* can only be *L* − 1. 

The distance between the projection of the testing sample *y* and the training samples with *W*_*m*_ in the new feature space is calculated as

(20) Di=||WmTy−WmTxi||2=||WmT(y−xi)||2 (i=1,2,…,n).

If the sample *x*_*k*_'s projection into the feature space has a minimum distance from the projection of the testing sample *y*, the testing sample will be classified into the same class as *x*_*k*_, such that

(21) $$\begin{array}{c} D_{k}=\min D_{i}<T\;\left(k,i=1,2,\ldots ,n;\;T\in \left[0,+\infty \right)\right), \end{array}$$

where *T* is a threshold to screen out the outliers. For the threshold LDA method, all the target members' projection distance *D*_*i*_ must be less than *T*, or otherwise they will be classified as outliers and rejected.

## 4. Experimental Results

In this experiment, we test the performance of the T-TPTSR, the T-GR, the T-PCA, and the T-LDA methods for outlier rejection and member classification, respectively. One of the measurement criteria for comparing the performance of these methods is to find the minimum overall classification error rate. During the classification task, an optimal threshold *T* can be found for the above methods so that the overall classification error rate is minimized. The overall classification error rate is calculated based on three classification error rates, such as the misclassifications among member's classes (when the testing sample is a member and *D*_*k*_ < *T*, but misclassified as another class), the misclassifications of a member to outlier's group (when the testing sample is a member but *D*_*k*_ > *T*, and thus misclassified), and misclassifications for outliers (when the testing sample is an outlier but *D*_*k*_ < *T*, and therefore accepted wrongly as a member). If ERR_overall_(*T*) represents the overall classification error rate as a function of the threshold *T*, ERR_member_(*T*) denotes the classification error rate for errors that occurred among members (misclassifications recorded for testing samples from member's group versus the total number of testing samples from member's group), and ERR_outlier_(*T*) is the misclassification rate for outliers (classification errors recorded for testing samples from the outlier's group versus the total number of testing outliers), their relationship can be written as

(22) $$\begin{array}{c} \text{ER}{\text{R}}_{\text{overall}}\left(T\right)=\text{ER}{\text{R}}_{\text{member}}\left(T\right)+\text{ER}{\text{R}}_{\text{outlier}}\left(T\right). \end{array}$$

It is noted that the value of ERR_member_ varies with the threshold *T*, and when *T* = 0, ERR_member_ takes the value of 100%, and it generally decreases when the value of *T* increases until it reaches a constant classification error rate. The classification error rate for outlier also changes its value according to the threshold *T*, however, ERR_outlier_ = 0% when *T* = 0, and its value increases until reaching 100%. The minimum ERR_overall_(*T*) can be found between the range of *T* = 0 and *T* = *T*_*m*_, where ERR_member_(*T*) becomes a constant, or ERR_overall_(*T*) reaches 100%, such that

(23) $$\begin{array}{c} \text{ER}{\text{R}}_{\text{opt}}=\min\;\text{ER}{\text{R}}_{\text{overall}}\left(T\right),\;T\in \left[0,+\infty \right). \end{array}$$

The value of ERR_opt_ is an important criterion showing the performance of a classifier for both of outlier rejection and member recognition.

Another measuring criterion for measuring the performance of the thresholded classifiers is the receiver operation characteristics (ROC) curve, which is a graphical plot of the true positive rate (TPR) versus the threshold *T* in the application of thresholded classification for outlier rejection. We firstly define the true positive detection rate for the outliers, TPR_outlier_(*T*), and it can be written in the form of the classification error rate for the outliers, such that

(24) $$\begin{array}{c} \text{TP}{\text{R}}_{\text{outlier}}\left(T\right)=100\%-\text{ER}{\text{R}}_{\text{outlier}}\left(T\right),\;T\in \left[0,+\infty \right). \end{array}$$

We also define the false alarm rate caused in the member' group as a function of the threshold, ERR_FA_(*T*), which is the number of errors recorded for misclassifying a member to an outlier versus the number of testing samples from the member's group. An optimal classifier for outlier rejection and member classification needs to find a suitable threshold *T* so that the TPR_outlier_(*T*) can be maximized as well as the ERR_FA_(*T*) can be minimized. Therefore, the following function *D*_*O*-*F*_(*T*) is defined for this measurement, such that

(25) DO-F(T)=TPRoutlier(T)−ERRFA(T)=100%−ERRoutlier(T) −ERRFA(T), T∈[0,+∞).

It is obvious that *D*_*O*-*F*_(*T*) is required to be maximized so that a classifier can be optimized for both outlier rejection and member classification, such that

(26) $$\begin{array}{c} D_{\text{opt}}=\max D_{O\text{-}F}\left(T\right),\;T\in \left[0,+\infty \right), \end{array}$$

and the value of *D*_opt_ is an important metric for comparing the performance of classifier for outlier rejection analysis.

The minimum overall classification error rates ERR_opt_ and the maximum difference of the true positive outlier recognition rate and the false-alarm rate *D*_opt_ are essentially the same performance assessment metric for a classifier with outlier rejection. The difference is that the overall classification error rate represents the efficiency of member classification, while *D*_*O*-*F*_ and *D*_opt_ show the performance of outlier rejection. In the following experiment, we test and compare the minimum overall classification error rates ERR_opt_ and the maximum *D*_opt_ of the T-TPTSR, T-GR, T-PCA, and T-LDA methods, respectively, and based on these two criteria we find the optimal classifier for outlier rejection and member classification.

In our experiment, we test and compare the performance of the above methods using the online face image databases Feret [30, 31], ORL [32], and AR [33], respectively. These databases provide face images from different faces with different facial expression and facial details under different lighting conditions. The Feret database provides 1400 face images from 200 individuals for the training and testing, and there are 7 face images from each of the classes. In the AR database, there are totally 3120 face images from 120 people, each of which provides 26 different facial details. For the ORL database, there are 400 face images from 40 different individuals, each of which has 10 face images.

In this experiment, the training set and the testing set are selected randomly from each of the individuals. For each of the databases, the people included are divided into two groups and one is member's group and the other is outlier's group. For individuals chosen as the member's class, the training samples are prepared by selecting some of their images from the database, and the rest of the images are taken as the testing set. For the outliers that is supposed to be outside the member's group, there is no training set for the classification, and all the samples included in the outlier's group are taken as the testing set. 

**Figure 1:**
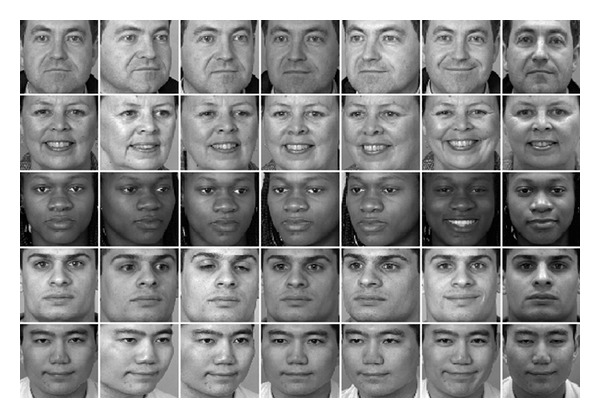
Part of the face images from the Feret database for testing.

We firstly test the Feret database with the above outlier rejection methods. The Feret database is divided into two groups, 100 members from the 200 individuals are randomly selected into the member's group, and the rest of the 100 individuals are the outliers in the test. For each of the 100 member classes, 4 images out of 7 are selected randomly as the training set, and the rest of the 3 images are for the testing set. For the 100 individuals in the outlier's group, all 7 images from each of them are the testing set for the classification task. Therefore, there are 400 training images and 1000 testing images in this test, and, among the testing images, there are 300 images from member's group and 700 images from outlier's group.  Figure 1 shows part of the member and outlier's images from the Feret database for the testing, and all the images have been resized to a 40 × 40-pixel image by using a downsampling algorithm [34]. Since the number of classes in the Feret database is much more than the ORL and AR databases, also the number of training images is less, and the resolution of the images is lower, the testing with the Feret database would be more challenging and the result is generally regarded as more convincing.

**Figure 2:**
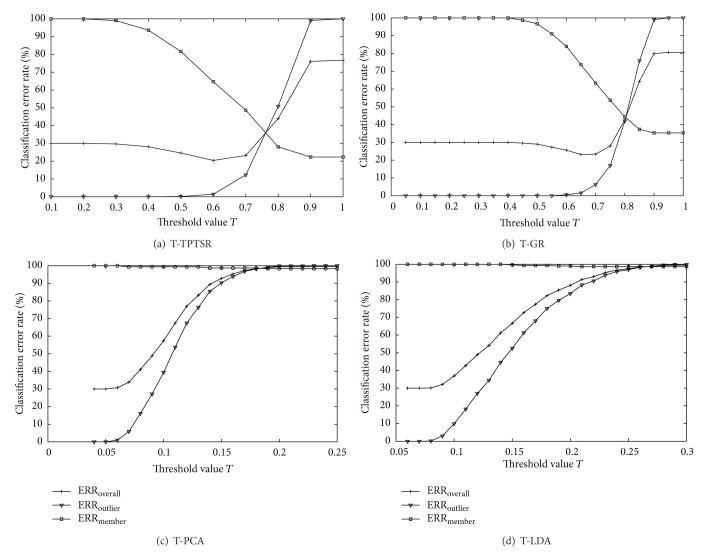
Classification error rates for outliers, members, and overall of (a) the T-TPTSR method, (b) the T-GR method, (c) the T-PCA method, and (d) the T-LDA method, respectively, on the Feret database.

**Figure 3:**
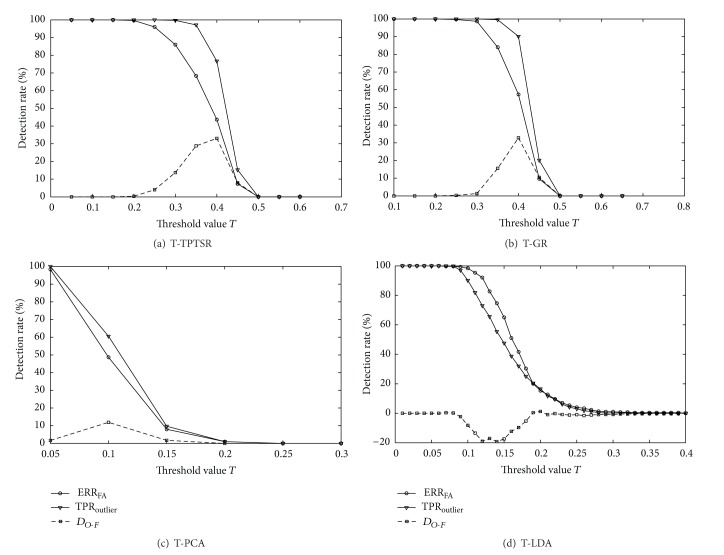
ROC curves for (a) T-TPTSR method, (b) T-GR method, (c) T-PCA method, and (d) T-LDA method, respectively, on the Feret database.

In the test of the T-TPTSR method with the Feret database, the number of nearest neighbors *M* selected for the first-phase processing is 60 (according to the empirical data, the optimal number *M* is selected about 10~15% of the number of training samples). In the test with the above methods, the threshold value *T* varies from 0 to a constant that can result in 100% of ERR_outlier_ with the interval of 0.1 or 0.5, where all outliers are accepted as members. Figures 2(a)~2(d) show different classification error rates of the above methods as the function of the threshold *T*, respectively. It can be seen that the ERR_opt_ values of the T-TPTSR method and the T-GR method are much lower than the T-PCA and T-LDA methods, and the ERR_member_ curves of the T-TPTSR and T-GR decrease from 100% to a much lower constant than those of the T-PCA and T-LDA when the threshold *T* increases. The second row of Table 1 lists all the ERR_opt_ values shown in Figure 2, and we can see that the T-TPTSR method achieves the lowest overall classification error rate.  Figure 3 shows the ROC curves of the T-TPTSR, T-GR, T-PCA and T-LDA methods, respectively, and the third row of Table 1 gives details of all the *D*_opt_ values shown in Figure 3. It can be seen that the T-TPTSR also has a higher value of *D*_opt_ than other methods.

**Figure 4:**
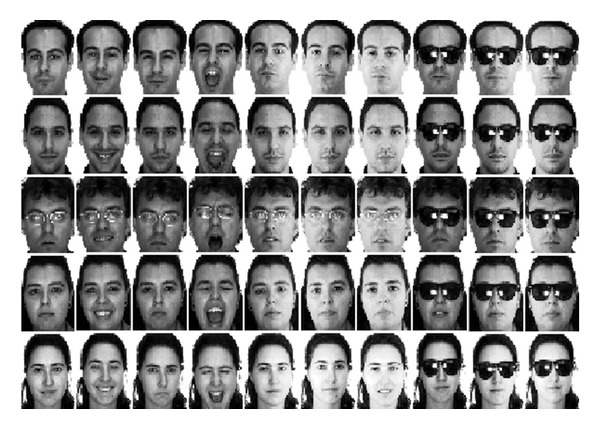
Part of the face images from the AR database for testing.

For the testing with the AR database, we randomly selected 80 classes as the member and the rest of the 40 people are taken as outliers. For each of the members, 13 images are selected randomly from the 26 images as the training set, and the rest of the 13 images are included in the testing set. Hence, there are 1040 training images and 2080 testing images in this test, and in the testing set, there are 1040 member's images and 1040 outlier's images.  Figure 4 shows part of the member's and outlier's images from the AR database, and the images for training and testing have been downsized to be a 40 × 50-pixel image [34]. 

When we test the T-TPTSR method with the AR database, the number of nearest neighbors *M* selected is 150.  Table 2 describes the ERR_opt_ values and *D*_opt_ values of the T-TPTSR, T-GR, T-PCA, and T-LDA methods, respectively, when tested with the AR database. It is obvious from the ERR_opt_ values and *D*_opt_ values that the T-TPTSR method outperforms the T-GR, the T-PCA, and the T-LDA methods in the outlier rejection and member classification applications.

**Figure 5:**
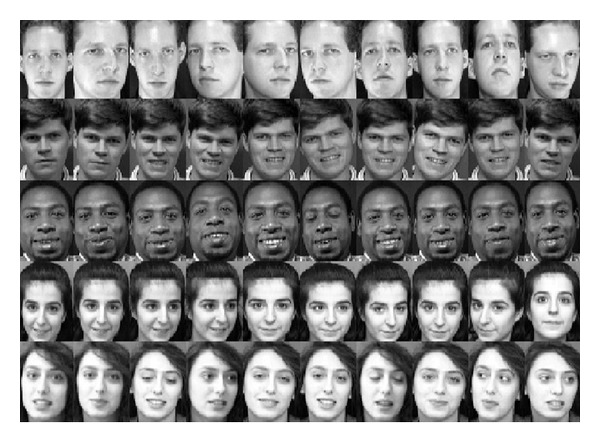
Part of the face images from the ORL database for testing.

We also test the above methods with the ORL face image database. There are totally 40 classes in the ORL database, and we select 30 random classes to be the members and the other 10 individuals to be the outliers. Among the 30 members, 5 images out of 10 for each of the members are selected randomly as the training samples, and the rest of the 5 images are the testing samples. So in the test, we have 150 training images and 250 testing images, and, in the testing set, there are 150 member's images and 100 outlier's images.  Figure 5 shows some sample images from the ORL database, and the images used are also resized to 46 × 56 [34]. 

The number of nearest neighbors selected for the T-TPTSR method for the ORL database is 40.  Table 3 gives the details of the ERR_opt_ values and *D*_opt_ values of the four methods, respectively. It can be seen that the T-TPTSR method also shows better performance than all the T-GR, T-PCA, and T-LDA methods, and it has been confirmed that the T-TPTSR method is the optimal solution among them for outlier rejection and member classification.

It is noted that, in the test with the AR and ORL databases, the performance of the T-TPTSR, the T-GR, and the T-PCA are comparable. This is because, under redundant and reasonable resolution sample situation, the performance of the T-PCA method is close to the T-TPTSR and T-GR methods. However, when the T-PCA method is tested with a small number of training samples and low-resolution images, like the Feret database, the advantages of the T-TPTSR method are very obvious. 

The criterion we use for judging, whether a sample is an outlier or not, is to measure the distance between the testing sample and the selected target class. If this distance is greater than the threshold, this sample will be classified as an outlier. In T-TPTPR method, the first-phase process finds a local distribution close to the testing sample in the wide sample space by selecting *M*-nearest samples. In the second-phase processing of the T-TPTSR method, the testing sample is classified based on the distance between the testing sample and the closest class among the *M*-nearest neighbors. If the testing sample is an outlier, the measure of distance will only be limited within the local distribution within the sample space, and, therefore, the measurement is not confused with other training samples that happen to be close to the outlier.

By applying a suitable threshold, a classifier can reject the outliers and classify the members with the minimum overall classification error rate and the maximum gap between the outlier detection rate and false alarm rate for members. The T-TPTSR method linearly representing the testing sample with the training samples and the distance between the testing sample and the target class are measured by calculating the difference between the testing sample and the weighted contribution of the class in the linear representation. In our test above, the T-TPTSR method achieves the best performance in outlier rejection as well as member classification. This is because in the T-TPTSR the two-phase linear representation of the testing sample results in a closer approximation and assessment by the training samples. Thus, the distance between the testing sample and the target class can be minimized, and the distance between the testing sample and an outlier can be maximized, leading to a better overall classification rate and greater ratio of outlier recognition rate versus the false alarm rate.

## 5. Conclusion

This paper introduces the modified versions of four useful approaches in face recognition, the T-TPTSR method, the T-GR method, the T-PCA method, and the T-LDA method, for the application of outlier rejection as well as member classification. Their performance is tested with three different online face image databases, the Feret, AR, and ORL databases, respectively. The results show that the T-TPTSR method achieves the lowest overall classification error rate as well as the greatest difference between the outlier detection rate and false-alarm rate. Even the T-PCA method may achieve comparable performance with the T-TPTSR method under ideal sample conditions, the test result of the T-PCA method is generally poor under bad sample conditions. The T-TPTSR method achieves the best performance in outlier rejection as well as member classification because of the two-phase linear representation of the testing sample with the training samples.

## Figures and Tables

**Table 1:** Minimum overall classification error rate and maximum ROC difference for T-TPSR, T-GR, T-PCA, and T-LDA methods, respectively, on the Feret database.

Methods	T-TPTSR	T-GR	T-PCA(150)	T-LDA(149)
ERR_opt_ (%)	20.4	23.2	30.0	30.0
*D*_opt_ (%)	33.0	32.8	11.9	1.24

T-PCA(150) indicate that the T-PCA used 150 transform axes for feature extraction, and T-LDA(119) means that the T-LDA used 119 transform axes for feature extraction. Tables 2 and 3 show the method and number of transform axes used in the same way.

**Table 2:** Minimum overall classification error rate and maximum ROC difference for T-TPSR, T-GR, T-PCA, and T-LDA methods, respectively, on the AR database.

Methods	T-TPTSR	T-GR	T-PCA(1040)	T-LDA(79)
ERR_opt_ (%)	27.2	30.2	33.0	50.0
*D*_opt_ (%)	45.5	41.8	43.4	21.8

**Table 3:** Minimum overall classification error rate and maximum ROC difference for T-TPSR, T-GR, T-PCA, and T-LDA methods, respectively, on the ORL database.

	T-TPTSR	T-GR	T-PCA(200)	T-LDA(29)
ERR_opt_ (%)	21.2	24.0	22.8	60.0
*D*_opt_ (%)	58.6	57.3	57.3	30.0
